# Reduced Estradiol-Induced Vasodilation and Poly-(ADP-Ribose) Polymerase (PARP) Activity in the Aortas of Rats with Experimental Polycystic Ovary Syndrome (PCOS)

**DOI:** 10.1371/journal.pone.0055589

**Published:** 2013-03-26

**Authors:** Gabriella Masszi, Eszter Maria Horvath, Robert Tarszabo, Rita Benko, Agnes Novak, Anna Buday, Anna-Maria Tokes, Gyorgy L. Nadasy, Peter Hamar, Zoltán Benyó, Szabolcs Varbiro

**Affiliations:** 1 Cardiology Unit, Bajcsy-Zsilinszky Hospital, Budapest, Hungary; 2 Institute of Human Physiology and Clinical Experimental Research, Semmelweis University, Budapest, Hungary; 3 2^nd^ Department Obstetrical Gynecology Semmelweis University, Budapest, Hungary; 4 Department of Pathophysiology, Semmelweis University, Budapest, Hungary; 5 Department of Pathology, Semmelweis University, Budapest, Hungary; University of Southampton, United Kingdom

## Abstract

Polycystic ovary syndrome (PCOS) is a complex endocrine disorder characterized by hyperandrogenism and insulin resistance, both of which have been connected to atherosclerosis. Indeed, an increased risk of clinical manifestations of arterial vascular diseases has been described in PCOS. On the other hand endothelial dysfunction can be detected early on, before atherosclerosis develops. Thus we assumed that vascular dysfunction is also related directly to the hormonal imbalance rather than to its metabolic consequences. To detect early functional changes, we applied a novel rodent model of PCOS: rats were either sham operated or hyperandrogenism was achieved by implanting subcutaneous pellets of dihydrotestosterone (DHT). After ten weeks, myograph measurements were performed on isolated aortic rings. Previously we described an increased contractility to norepinephrine (NE). Here we found a reduced immediate relaxation to estradiol treatment in pre-contracted aortic rings from hyperandrogenic rats. Although the administration of vitamin D_3_ along with DHT reduced responsiveness to NE, it did not restore relaxation to estradiol. Poly-(ADP-ribose) polymerase (PARP) activity was assessed by poly-ADP-ribose immunostaining. Increased PAR staining in ovaries and circulating leukocytes from DHT rats showed enhanced DNA damage, which was reduced by concomitant vitamin D_3_ treatment. Surprisingly, PAR staining was reduced in both the endothelium and vascular smooth muscle cells of the aorta rings from hyperandrogenic rats. Thus in the early phase of PCOS, vascular tone is already shifted towards vasoconstriction, characterized by reduced vasorelaxation and vascular dysfunction is concomitant with altered PARP activity. Based on our findings, PARP inhibitors might have a future perspective in restoring metabolic disorders in PCOS.

## Introduction

Polycystic ovary syndrome (PCOS) is the most common endocrine disorder, affecting 4–11% of women. The lifelong disease often remains hidden because of its multidisciplinarity. The main problem is infertility but the disease influences the whole body in many different ways [1]. In complete fenotype PCOS is manifested with hyperandrogenism, ovulatory dysfunction and polycystic ovaries. Two of these are the diagnostic criteria for PCOS as the Rotterdam 2003 criteria defined it. As a metabolic component insulin resistance plays role in the disorder and obesity accompanies it in 50–60% of the cases. These two with other developing risk factors such as metabolic syndrome, hypertension and diabetes mellitus together put women with PCOS at a high risk for cardiovascular diseases [2]. This was proved by Christian et al. in 2003, when coronary artery calcium, a marker for coronary arteriosclerosis, was measured by electron beam computed tomography in women with PCOS and without PCOS at similar age. The results showed PCOS women were increased risk for atherosclerosis as coronary artery calcium was more prevalent in them then in control group from obesity [3].

Indeed, haemodynamic changes of internal carotid artery [4] and early predictors of endothelium dysfunction in women with PCOS were detected, as FMD (flow mediated dilatation) and nitrate-mediated dilation were significantly lower in women with PCOS than in the control group [5]. Moreover, a recent meta-analysis found a two-fold risk of coronary heart disease and stroke for patients with PCOS compared to women without PCOS [6].

Estrogens have a multiple cardiovascular protective effect. These include both a rapid and a long-term vasorelaxing effect, the latter of which depends on gene expression and employs different signalling pathways not as in short term effects [7],[8]. In PCOS either the relative or the absolute absence of estrogens might contribute to the higher cardiovascular risk.

Vitamin D was effective in PCOS: a daily intake of vitamin D reduced obesity in PCOS patients [9], however oral vitamin D treatment did not improve insulin resistance [10].

Poly-(ADP-ribose) polymerase-1 (PARP-1) is a ubiquitously expressed nuclear enzyme, which has a central role in responses to cellular injury [11]. PARP-1 is activated by the presence of single strand DNA. PARP-1 cleaves NAD into nicotinamide and ADP-ribose, and attaches polymers of the latter product to nuclear acceptor proteins including histones, transcription factors, and the PARP itself. Through PARylation PARP regulate DNA repair by attracting DNA ligase III to the DNA. On the other hand, overactivation of PARP consumes NAD, resulting in decreased glycolysis, electron transport and ATP formation. While the activation of PARP-1 by limited, sub-lethal injury may facilitate DNA repair and cell survival, irreparable DNA damage triggers – either apoptotic or necrotic – cell death [11].

This signalling pathway has been implicated in both experimental models and in human cardiovascular diseases [12], [13]. PAR polymerisation has been proved in several clinical settings; in patients suffering from myocardial infarction and therapeutic revascularization, PARP activation was detected in circulating leukocytes [14], [15]. Activation of PARP was also demonstrated in the failing hearts by an increased abundance of poly-ADP ribosylated proteins when immunohistochemical analysis revealed that PARP activation was localized to the nucleus of the cardiomyocytes from the failing hearts [16]. In different rodent models of diabetic cardiomyopathy, a significant increase in (poly-ADP)-ribosylation was detected in cardiac myocites and endothelial cells, which were remarkably improved by PARP inhibitors [17], [18]. Poly(ADP-ribose polymerase-1 regulation was detected in the progression of autoimmune nephritis by inducing necrotic cell death and modulating inflammation [19]. In a phase II prospective, single-blind, multi-centre, dose escalation study of a single dose of intravenous PARP inhibitor (INO-1001) (200 mg, 400 mg, or 800 mg) was administered to 30 patients between the ages of 48 and 63 years with acute ST-segment elevation myocardial infarction (STEMI), who were to be treated with primary percutaneous coronary intervention (PCI). The PARP inhibitor INO-1001 was found to induce a tendency to reduce the plasma levels of C-reactive protein and the inflammatory marker IL-6, No drug-related serious adverse events were observed in the patients receiving the drug during the study period [20]. Based on the above mentioned facts, we hypothesized that PARP-1 may be activated in PCOS as well.

Besides the available pharmacological inhibitors, there is cumulating amount of evidence for endogenous modulators, too. Recently the active forms of vitamin D_3_ has been shown to inhibit PARP by Mabley et al., it was demonstrated that UV irradiation-mediated PARP activation in human keratinocytes can be inhibited by treatment with vitamin D, 7-dehydrocholesterol or 1alpha,25-dihydroxyvitamin D_3_ [21]. Interestingly, a gender difference was described in the PARP-1 activity, Mabley et al. [22] showed that gender specific inflammatory response is preferencially downregulated by PARP in male but not in female animals. This was proven in stroke, as McCullogh found that PARP inhibition and PARP-1 deficiency conferred their protection only in male mice, they had no benefit to the female ones in cerebral ischaemic damage [23].

The same group examined the downstream mediators of NO/PARP activation to investigate possible mediators of ischemic sexual dimorphism. Apoptosis inducing factor translocation and PAR formation did not mediate ischemic injury in the female mice brain; although equivalent activation of cell death pathway occurred in both sexes after ischemia, detrimental effects were only present in males [24].

The aim of this study was to show altered regulation of vascular tone in PCOS applying a novel rodent model. We wanted to detect the possible beneficial effect of chronic vitamin D administration and acute estradiol treatment. Also we aimed to look at altered DHT induced PAR response in different organs and sought to identify a correlation between the changes in vascular reactivity and PAR responses.

## Methods

**Ethics approval** The investigation conforms to the Guide for the Care and Use of Laboratory Animals published by the US National Institutes of Health and was approved by Semmelweis University Animal Care Commission (IRB approval: 22.1/2960/003/2009).

### Animals

The investigation conforms to the Guide for the Care and Use of Laboratory Animals published by the US National Institutes of Health and was approved by Semmelweis University Animal Care Commission (IRB approval: 22.1/2960/003/2009) conforming to the Institutional Animal Care and Use Committee (IACUC). Thirty adolescent, 21–28 day-old, female Wistar rats, weighing 100–140 g were purchased from Semmelweis University Animal Colony (Budapest, Hungary, originated from Charles River Ltd). The animals were randomized into 3 treatment groups: control, DHT and DHT+vitamin D_3_. No medical or surgical complications were observed throughout the whole period. Conventional rat chow and tap water were provided ad libitum. All surgery was performed under sodium pentobarbital anesthesia, and all efforts were made to minimize suffering.

### Experimental PCOS model and the treatment procedures

To induce experimental PCOS, twenty animals received 70-day-long continuous-release subcutaneous pellets of 7.5 mg dihydro-testosterone (DHT, Innovative Research of America, Sarasota, Fl, USA) underneath the back skin, releasing a daily dose of 83 μg DHT as described by Manneras et al. [25]. Rats were anesthetized with 45 mg/kg sodium pentobarbital (Nembutal, Phylaxia-Sanofi, Budapest, Hungary). Ten animals underwent sham operations (control group). Following the chronic surgical interventions, 20 mg amoxicillin +4 mg clavulanic acid combination (Augmentin GlaxoSmithKline, Memphis, USA) was administered intramuscularly. Ten animals from the DHT group received subcutaneously 120 ng/100 g body weight 1,25-dihydroxy-vitamin D_3_ (DHT+ D_3_ group, Inj. Calcijex, 2 μg/ml, Abbott Lab., Illinois, USA) every week, which is a slight modification of the protocol previously described by Przybylski et al. [26]. Our choice of weekly administration was preferred instead of the previously described daily injections to minimize stress. Animals in the control and the DHT group received vehicle subcutaneously (saline). Chronic treatments were continued for 70 days before the conclusion of the experiments with ex vivo measurements.

### Chemicals and reagents

Normal Krebs-Ringer (nKR) solution was composed of (in mM): 119 NaCl, 4.7 KCl, 2.5 CaCl_2_, 1.17 MgSO_4_, 20 NaHCO_3_, 1.18 KH_2_PO_4_, 0.027 EDTA, and 11 glucose (Sigma Aldrich St. Louis, MO, USA and Budapest, Hungary). Hyperkalaemic solution was prepared from normal KR. It was supplemented with KCl to achieve 124 mM potassium level. Temperature of the solution was kept at 37°C, and it was aerated with a 5% CO2/95% O2 mix (Lindegas, Répcelak, Hungary). Norepinephrine and 17- estradiol were obtained from Sigma-Aldrich. Drugs were freshly prepared in nKR solution on the day of the experiment.

### Ex vivo pharmacological reactivity of thoracic aorta rings

After 70 days of treatment, rats were anesthetized. For anesthesia we used penthobarbital (Nembutal, Phylaxia-Sanofi, Budapest, Hungary) 45 mg/kg intraperitoneally. Animals were perfused transcardially with 10 ml heparinized (10 IU/ml) nKR solution. The aorta of each animal was then removed. The distal part of the thoracic aorta (TA) was isolated and four rings were prepared and placed into a vessel chamber filled with nKR solution aerated with 95% O2/5% CO2 mix. Thoracic aorta segments of 3 mm length from each experimental group were mounted on stainless steel vessel holders (200 µm in diameter) of a conventional myograph setup (610-M Multi Myograph System; Danish Myo Technology, Aarhus, Denmark). The organ chambers of the myographs were filled with 8 ml of nKR solution. The bath was warmed to 37°C, and the resting tension of TA rings was adjusted to 15 mN, according to previous studies [27].

Segments were exposed to 124 mM K^+^ to elicit a reference contraction. Twenty minutes later, precontraction was induced by norepinephrine (5×10^−8^ M) and for estradiol-mediated vasorelaxation cumulative dose-response measurements were used (10^−5^–10^−4^ M). Norepinephrine contraction was expressed as a percentage of K^+^-precontraction. Aortic relaxations were tested after a stable plateau of contraction had been reached. Relaxant responses were expressed as a percentage of the precontraction produced by norepinephrine (percentage =  (tension after norepinephrine-tension after estradiol)/(tension after norepinephrine-baseline tension) * 100). The isometric tension recording of the thoracic aorta segments was made with the MP100 system, and recorded data were analyzed with AcqKnowledge 3.7.3 software (BIOPAC Systems, Goleta, CA). Vasoactive substances were dissolved in nKR. All concentrations are expressed as the final concentration in the organ bath.

### Histology

Ovaries and aorta rings of the animals were collected and freshly fixed for histological examinations. Tissue samples were immersion fixed in 4% buffered formaldehyde, hematoxylin-eosin staining was prepared and specimens were examined using a Zeiss AxioImager. A1 microscope coupled with Zeiss AxioCAm MRc5 CCD camera for evaluation Pannoramic viewer software was used; 3DHISTECH Ltd., Budapest, Hungary.

### Immunohistochemistry

Leucocytes were isolated from venous blood samples using Histopaque-1083 (Sigma Aldrich, St Louis, MO, USA). Methanol-fixed smears were prepared from the cell suspension. Samples of ovaries and aortas were fixed with formalin, embedded in paraffin and 5 μm thick sections were prepared.

After deparaffinization and antigen retrieval (0.1 mmol/l citrate buffer, pH 3, heated in a microwave oven for 15 min), samples were incubated with a mouse monoclonal anti-poly-ADP-ribose (PAR) antibody overnight at 4°C (Calbiochem, San Diego, CA, USA, 1∶1,000). Secondary labelling was achieved using biotinylated anti-mouse horse antibody (Vector Laboratories, Burlingame, CA, USA) (30 min, room temperature). Horseradish peroxidase-conjugated avidin (30 min, room temperature) and nickel-enhanced diaminobenzidine (6 min, room temperature, black color) was used (Vector Laboratories). Tissue sections and smears were counterstained with nuclear fast red.

To evaluate PAR-staining, the following semiquantitative score system was applied by a blinded observer as described previously [28], [29]. The semiquantitive evaluation scoring system was: 1 point: no staining; 2 points: light cytoplasmic staining; 3 points: strong cytoplasmic staining; 4 points: cytoplasmic staining with a few positive nuclei; 5 points: approximately 50% of the nuclei positive; 6 points: approximately 75% of the nuclei positive; 7 points: general nuclear staining with a few negative cells; 8 points: all nuclei positive; 9 points: strong nuclear staining in all cells; 10 points: very strong general nuclear staining in all cells. In case of leukocyte smears 5 fields were evaluated with a minimum of 300 cells. In case of arteries and ovaries, the whole tissue section was examined.

### Statistical analysis

Parametric variables were analyzed by applying ANOVA. As a post hoc test, Newman-Keuls` test was applied. P<0.05 was uniformly accepted as a significant difference. Data were presented as mean ± SEM.

## Results

### Acute estrogen treatment induces a weaker vasorelaxation in aortas from hyperadrogenic animals

**Figure 1:**
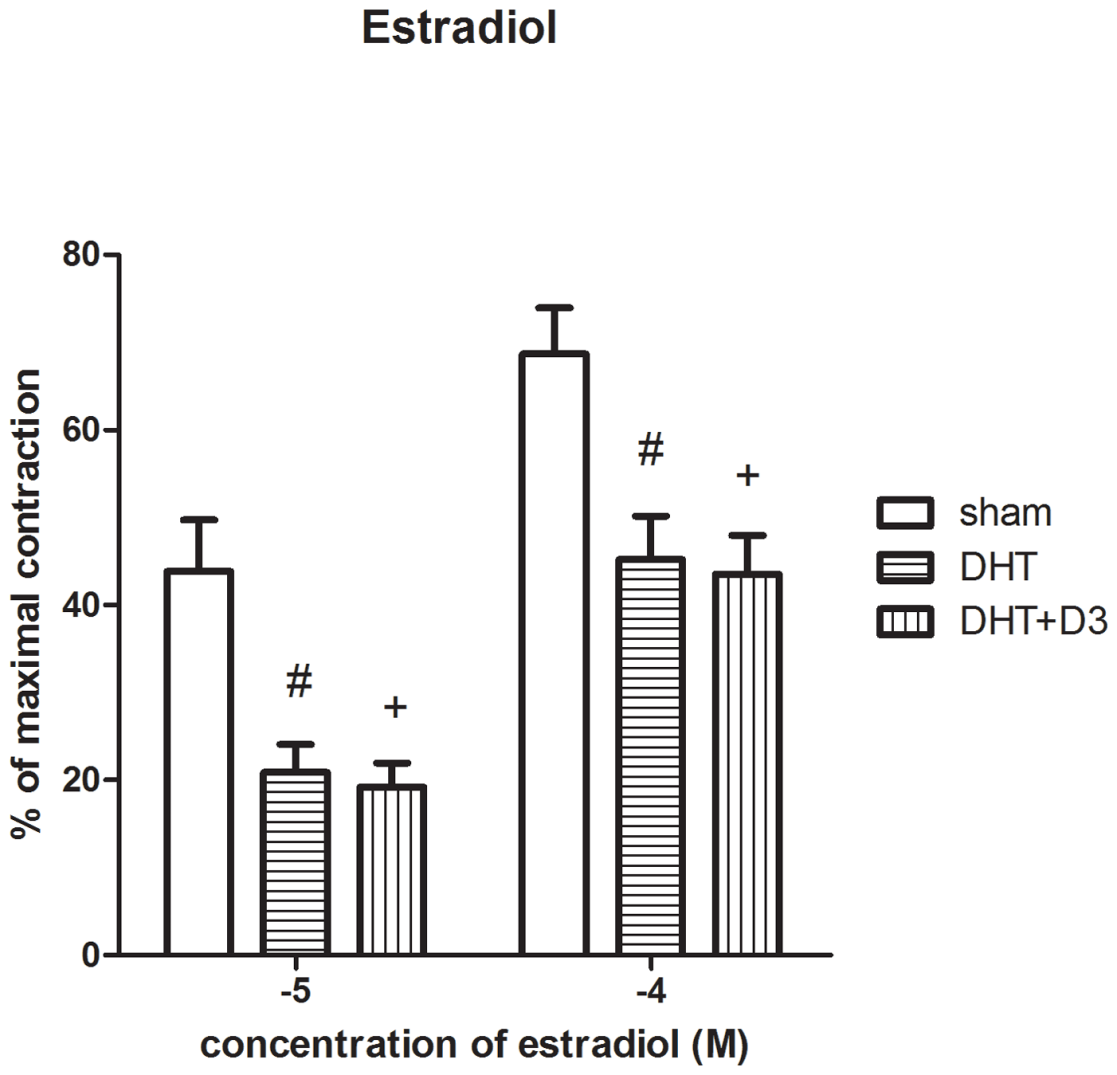
Estradiol dependent vasorelaxation is weaker in experimental PCOS. Rats were treated with DHT for 70 days +/− vitamin D_3_ when they were sacrificed and aorta rings were isolated for myography studies. Specimens were first precontracted with norepinephrine (5×10^−8^ M) and then treated with two different doses of estradiol as shown. Vasodilation is expressed as a percentage of difference between maximally contracted and maximally dilated tone of the vessels according to the following equation (percentage =  (tension after norepinephrine-tension after estradiol)/(tension after norepinephrine-baseline tension) * 100). The difference between control and DHT (# = p<0.01) or control vs. DHT+ D_3_ animals (+ = p<0.01) were both significant. The difference between DHT and DHT+ D_3_ was not significant.

**Figure 2:**
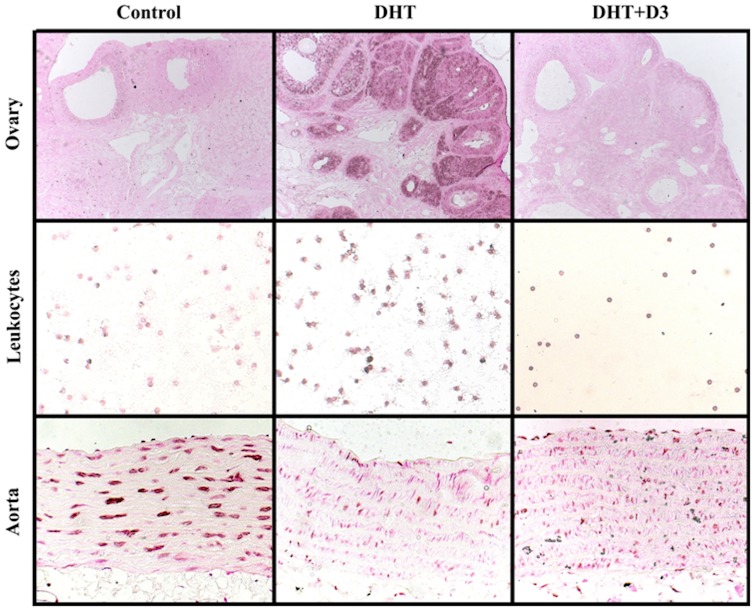
PAR staining is reduced in both the endothelium and the smooth muscle cells of aortas in PCOS. Ovaries, aortas and circulating leukocytes were isolated from animals exposed to the three treatments (control, DHT, DHT+ D_3_) as described previously. PAR immunohistochemistry was performed. Representative images were taken. PAR staining was augmented in ovaries and leukocytes, however was reduced in both the endothelial and the muscular layer of the aortas in the DHT animals. Vitamin D_3_ reduced the augmented staining detected in ovaries and leukocytes, but did not alter the staining in the aortas. It is notable that the DHT treatment induced the morphological changes typical in PCOS.

To examine the vascular dysfunction in the early phase of polycystic ovary syndrome (PCOS) we first treated rats with dihydrotestosterone (DHT) for 70 days as described by Manneras et al. [25]. A subgroup of animals was simultaneously treated with vitamin D_3_ (Figure 1). Histology of the ovaries from DHT-treated rats showed a polycystic morphology, which was not altered by the administration of vitamin D (see later in the Results section and Figure 2).

Ex vivo experiments were performed on excised aorta rings. As a control, the aorta rings were first challenged with hyperkalaemia (total extracellular potassium 124 mM) and showed similar contractility in all treatment groups. Since the relative hypoestrogenic state in PCOS is considered to contribute to the vascular dysfunction we asked whether acute estrogen treatment can restore normal vascular tone. To explore this, aorta rings were precontracted with norepinephrine (5×10^−8^ M) for approximately 15 minutes and then were treated with estradiol (E2: 10^−5^–10^−4^ M incubation approximately 25 minutes to let the signal transduction process work). Estradiol readily induced vasorelaxation in the precontracted aortas. Surprisingly this effect was significantly weaker in the DHT group compared to the control group. Vitamin D_3_ did not restore the vascular responsiveness to estradiol, namely E2 induced a similar relaxation in the DHT+vitamin D_3_ as in the DHT alone group. (Figure 1. Control vs. DHT+D_3_ p<0.05; DHT vs. DHT+D_3_ ns,). Thus short-term estrogen treatment is less potent as a vasorelaxing agent in large arteries from hyperandrogenic animals. In the rat aortas treated by dihydrotestoterone, the estradiol dependent vasorelaxation was substantially reduced (p<0.01) compared to the control group, and vitamin D_3_ treatment could not correct it (Figure 1).

### PARP activity is selectively regulated in different tissues in hyperandrogenic states

**Figure 3:**
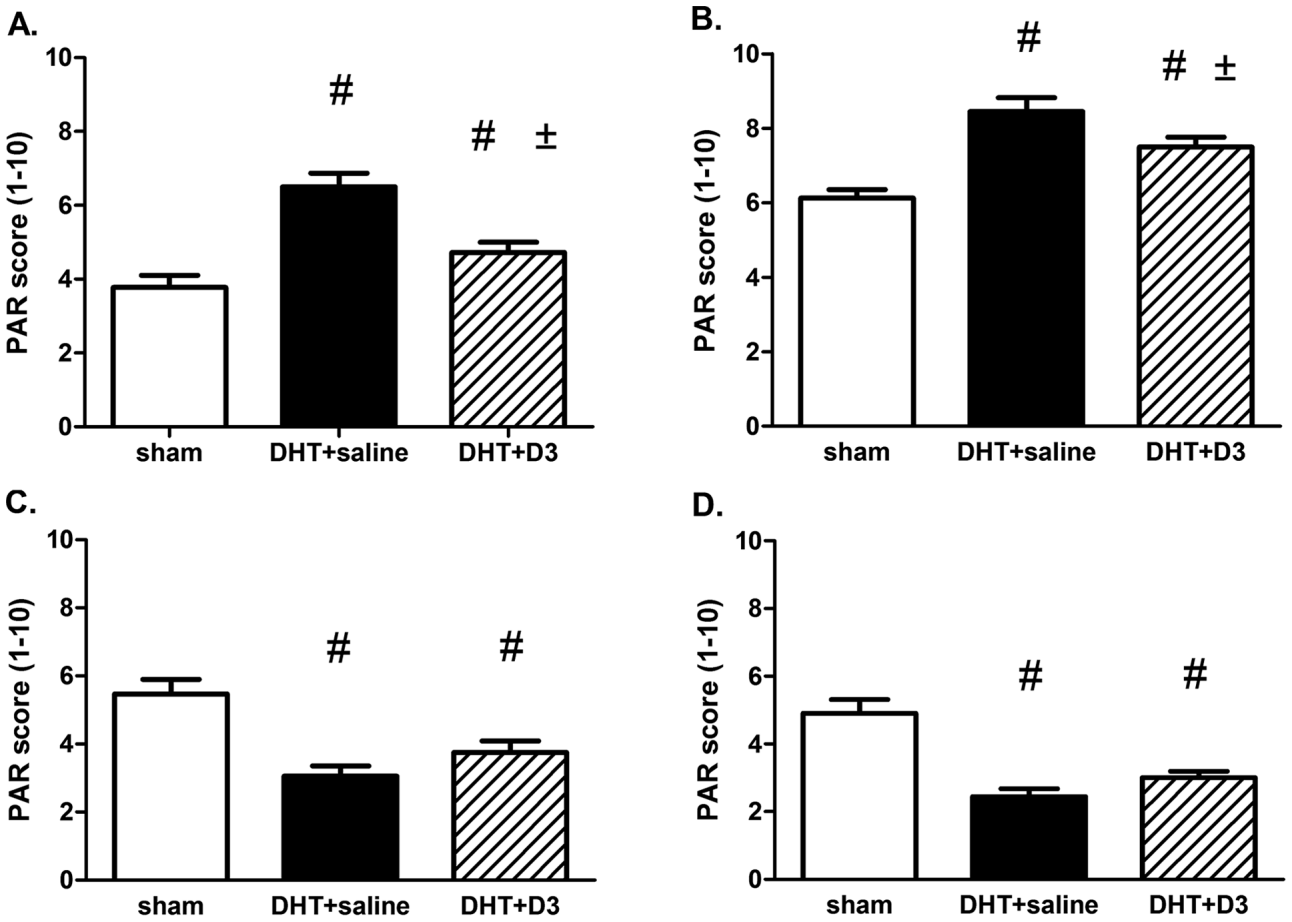
Statistical analysis confirmed the significant changes in PAR staining intensities in the three experimental groups. Slides from Figure 2. were evaluated using a 10-graded scale (PAR score) as described in Methods. Results from ovaries (A), circulating leukocytes (B), vascular endothelium (C) and vascular smooth muscle cells (D) are presented. Figure 3. A, B: Control vs. DHT (# = p<0.05), DHT vs. DHT+ D_3_ (± = p<0.05). Figure 3. C, D: Control vs. DHT, and Control vs. DHT+ D_3_ (# = p<0.05).

PARP activity is a marker of cellular DNA damage, and it was also shown to be controlled by estrogens [12]. PARP activity can be monitored by the amount of poly-ADP-ribosylated (PAR) proteins. Tissue samples from control, DHT and DHT+vitamin D_3_ groups were collected, histology and immunohistochemistry – using PAR-antibody – were performed. Samples were evaluated according to a 10-grade semiquantitive scale [29]. In control animals we found similar PAR staining in the different tissues – circulating leukocytes, ovaries, endothelium, vascular smooth muscle cells. When the different treatment groups were compared we have found two distinct patterns: the DHT treatment enhanced PARP-activity in the ovaries and the circulating leukocytes, which was inhibited by vitamin D_3_ (Figure 2. and Figure 3. A, B; Control vs. DHT p<0.05, DHT vs. DHT+D_3_ p<0.05,). On the contrary, in the vascular endothelium and smooth muscle cells the PAR staining in the DHT group was significantly weaker (Figure 2. and Figure 3. C, D; Control vs. DHT and Control vs. DHT+D_3_ p<0.05).

## Discussion

Our experiment has been the first to study the relaxing capacity of estradiol on the vessel wall under a hyperandrogenic milieu and the effect of vitamin D_3_ therapy on it in a recent rodent model of PCOS. Parallelly, we were detecting the DHT-induced PAR response, PAR poly(adenosinphosphate-ribose) accumulation and the effect of vitamin D_3_ therapy on it in different organs to assess the activation and inhibition of PARP enzyme in our PCOS simulating clinical circumstances.

In women with PCOS significant pharmacological reactivity changes develop as well as mechanical damages of the large blood vessels [3], [4]. The changes in smooth muscle and endothelium related relaxation, as well as vasoconstriction, were studied in PCOS and in hyperandrogenic circumstances. Kravariti measured a significant decrease in both endothel dependent (FMD), and smooth muscle related (nitrate mediated) relaxation compared to the normal controls [5].

Estrogen has a variety of effects on vasculature including the regulation of vascular tone. The vasodilatory effects of estrogen can be endothelium dependent and independent, and they can work by genomic and nongenomic mode of action [30]. Estrogen receptors are present in all components of the blood vessel wall: endothelium, smooth muscle and adventitia [31]. The vasodilatory capacity of estrogen is well documented both in vivo and in vitro. Andersen et al. differentiated the long term and acute vasomotor responses to estradiol in rat aorta: the long term effect was mediated through the endothelium mostly by increasing NO release, but the acute effect of estradiol seemed to be brought on through an effect of vascular smooth muscle cells. Developed contraction of different contractile agents had been reduced by the effect of both long-term and acutely given estradiol, but in varying degrees [32].

Testosterone also has a moderate vasorelaxant effect acutely, especially by the modulation of vascular smooth muscle ion channel function, particularly the inactivation of L- type voltage operated Ca^2+^ channels and/or activating voltage – operated Ca^2+^ and K^+^ channels [33]. Torres et al. investigated the modulation of aortic vascular reactivity by sex hormones in a male rat model of metabolic syndrome. He found that testosterone caused endothelial dysfunction and he detected a protective effect of castration, or of estradiol, given to the castrated animals [34]. There is no data in polycystic ovary syndrome and only a few items of data are available of the effects of estrogen on blood vessels in a hyperandrogenic milieu.

In our study we found an impaired vasorelaxant effect of estrogen. Athough it was still working, the vasorelaxant effect was substantially reduced in hyperandorgenic milieu caused by the PCOS (Figure 1). New's [35] results showed that the positive cardiovascular effects of estrogen do not manifest themselves in a hyperandrogenic medium. Compared to the control men group, there were no difference in arterial compliance, blood pressure, cholesterol levels and cardiovascular risk altogether in estradiol treated male-to-female transsexuals. Our results correlate with results of New's studies. Li et al. found that estrogen potentiates the constrictor prostanoid function in female rat aorta by upregulation of the cyclooxygenase-2 and thromboxane pathways both in the endothelium and vascular smooth muscle by upregulating the expression of thromboxan receptors in vascular smooth muscle comparing to males and ovariectomized females [36].

Our immunhistochemical analysis showed the expected DHT-induced PAR-response in the different tissues regarding ovary and leukocytes. The DHT-induced PAR-response increased in DHT treated animals and vitamine D_3_ therapy reduced it significantly. (Figure 3. A, B) But in the aorta, both in the endothelium or the vascular smooth muscle, the DHT induced PAR-response was significantly reduced by DHT treatment and vitamin D_3_ did not influence it any more (Figure 3. C, D). Similar regional difference was observed in the effect of insulin in PARP activity. Insulin lowered PARP activity in leukocytes, however, it did not influence the placenta and umbilical artery [28]. This regional difference might be the compensatory, protective effect of DHT – as described by Xu et al. on HUVEC culture. They found a decrease in the activity of oxydative stress enzymes, caspase-3 and -9 in endothelial cells, following DHT pretreatment [37].

There are emerging evidences about several endogeneous and exogeneous modulatory factors on PARP activity; including estrogen and the active forms of vitamin D as PARP inhibitor [38].

As it was detected in many clinical conditions but not in policystic ovary syndrome, we studied the PAR activation in a rodent model of PCOS. In PCOS the cardiovascular risk is elevated as unfavourable hormonal – hyperandrogenic, metabolic, prediabetic and inflammatory alterations are present from a younger age till menopause [39]. We also wanted to examine the inhibitory capacity of vitamin D_3_ on poly (ADP-ribose) polymerase compared to controls and DHT treated animals.

Our results were coherent with earlier studies in hypertension and diabetes mellitus [12], [13], [38], [40] as DHT treatment increased poly(ADP-ribolysation) in the ovarium and leukocytes in concordance with the clinical state of elevated risk of hypertension and diabetes mellitus in PCOS. Further studies are needed to clarify the role of PARP in developing hypertension, diabetes mellitus and cardiovascular damage in PCOS, but our study could be the first step in this direction.

To sum up, the activation of the poly(ADP-ribose) polymerase was shown in many different clinical and pathological settings (chronic inflammation, autoimmune diseases e.g.;arthritis, colitis), vascular diseases (atherosclerotic plaques, in microvessels in diabetic patients) ischaemic diseases (myocardial infarction, bypass surgery, stroke), and in chronic heart failure [13], but in PCOS it had not been examined before. In our measurements, the detected elevation of PARP activation in the leukocytes and the ovaries of polycystic ovary syndrome induced rats proved our hypothesis, as it was similar in other pathological conditions. On the contrary, DHT treatment significantly reduced PARP activity both the endothelial and smooth muscle layer of the aorta and Vitamin D_3_ treatment did not have any significant influence on this effect.

## Conclusions

The DHT treatment caused alterations in relaxant capacity. Estrogen in hyperandrogenic milieu could not work as well as in the control female rat aortic rings and the influence of vitamin D_3_ on them was not significant. This result might give the clue to one of the key-mechanisms of early cardiovascular damage in PCOS.

In PCOS, caused by DHT treatment, deleterious effects were exerted partly via PARP activity (ovaries and leukocytes), and these could be reversed by vitamin D. But in aortic walls, both in endothelium or vascular smooth muscle, the PARP activity was significantly reduced by DHT treatment and vitamin D_3_ did not influence it significantly. Similarities of PARP expression and estrogen dependent relaxation may raise the possibility of causal connections, which require further investigation.
