# Correction: Reduced Estradiol-Induced Vasodilation and Poly-(ADP-Ribose) Polymerase (PARP) Activity in the Aortas of Rats with Experimental Polycystic Ovary Syndrome (PCOS)

**DOI:** 10.1371/journal.pone.0091625

**Published:** 2014-02-28

**Authors:** The PLOS ONE Staff

Affiliation number 5 for the seventh author, Anna-Maria Tokes, is incorrect. The correct affiliation is: 2nd Department of Pathology, Semmelweis University, MTA-SE Tumour Progression Research Group, Budapest, Hungary.
